# Sex‐Based Differences in Symptomatology in the First Month Following Atrial Fibrillation Catheter Ablation

**DOI:** 10.1111/jce.70009

**Published:** 2025-07-11

**Authors:** Mark T. Mills, Gregory Y. H. Lip, Vishal Luther, Dhiraj Gupta

**Affiliations:** ^1^ Department of Cardiology Liverpool Heart and Chest Hospital Liverpool UK; ^2^ Liverpool Centre for Cardiovascular Science at University of Liverpool Liverpool John Moores University and Liverpool Heart and Chest Hospital Liverpool UK; ^3^ Department of Clinical Medicine Aalborg University Aalborg Denmark

**Keywords:** atrial fibrillation, catheter ablation, pulmonary vein isolation, quality of life, sex differences

## Abstract

**Background:**

Differences in baseline characteristics and clinical outcomes exist between female and male patients with atrial fibrillation (AF).

**Objective:**

To assess sex‐specific symptoms within 1 month of AF catheter ablation.

**Methods:**

Patients undergoing AF ablation between 2000 and 2024 were identified from 57 healthcare organizations using a global federated research network. Female and male patients were 1:1 propensity score matched (PSM) based on baseline characteristics. Symptoms within a month of ablation were identified using ICD‐10 codes and classified into major systems: cardiac (chest pain, palpitations), respiratory (dyspnea, cough), gastrointestinal (nausea, vomiting, heartburn, dysphagia, bloating, diarrhea, constipation, anorexia), neurological (headache, visual disturbance, speech disturbance, dizziness) and urological (urinary retention and dysuria).

**Results:**

After PSM, 69 244 patients were included (34 622 in each group). Female patients had a higher incidence of cardiac (female, 8.9% vs. male, 6.1%; *p* < 0.001), respiratory (7.9% vs. 6.1%; *p* < 0.001), gastrointestinal (3.4% vs. 2.2%; *p* < 0.001) and neurological symptoms (3.1% vs. 2.5%; *p* < 0.001) compared with male patients. Urological symptoms were more common in male patients (1.6% vs. 0.9%; *p* < 0.001) due to a higher incidence of urinary retention (1.1% vs. 0.3%; *p* < 0.001). All individual symptom components of cardiac, respiratory, gastrointestinal and neurological composites were more common in female patients, except from heartburn (0.1% vs. 0.1%; *p* = 0.49), bloating (0.2% vs. 0.2%; *p* > 0.99), anorexia (0.1% vs. 0.1%; *p* = 0.79), and speech disturbance (0.2% vs. 0.2%; *p* = 0.51) which were similar between sexes.

**Conclusion:**

Compared with male patients, female patients experience higher rates of cardiac, respiratory, gastrointestinal, and neurological symptoms within 1 month of AF ablation.

## 1 Introduction

Sex‐based differences in the presentation, management, and outcomes of patients with atrial fibrillation (AF) have long been recognized. Women with AF tend to be older at diagnosis, have a higher symptom burden and lower quality of life, and present with a greater prevalence of cardiovascular comorbidities (such as heart failure with preserved ejection fraction and hypertension) compared with men [1, 2, 3, 4]. Women are also, on average, less tolerant of antiarrhythmic drugs and referred less often— or later—for AF catheter ablation than men [5, 6, 7]. Further, studies examining the relationship between sex and clinical outcomes after AF ablation have yielded conflicting results; some report comparable short‐ and long‐term efficacy with similar quality‐of‐life improvements [8, 9], while others indicate lower efficacy and higher adverse events in women, including increased rates of vascular access‐related bleeding and pericardial effusion [10, 11].

Despite these well‐documented disparities, early postablation symptom patterns remain an underexplored aspect of AF management. As women with AF often report greater symptom severity pre‐procedure [12], it is possible that these symptoms may persist or evolve differently in the early recovery period and that higher comorbidity burden may further influence symptom perception and reporting. A clearer understanding of these differences could help in refining follow‐up strategies, enhancing symptom management, and ultimately improving procedural success and patient satisfaction across both sexes.

This study examines sex‐based differences in clinical symptoms during the first month following AF catheter ablation. By analyzing healthcare‐reported symptoms, we aim to identify distinct symptom patterns and potential disparities in postablation recovery.

## 2 Methods

### 2.1 Study Design, Setting, and Participants

We conducted a multicentre, retrospective cohort study using the TriNetX Research Network Database, a global, federated research platform providing real‐time access to healthcare record data from over 250 million patients across more than 120 global healthcare institutions in 19 countries spanning North and South America, Europe, Asia‐Pacific, the Middle East, and Africa [13, 14, 15]. The database includes deidentified patient data from both inpatient and outpatient settings, contributed by academic and community‐based healthcare organizations. To comply with legal and ethical frameworks and prevent data re‐identification, the identities of participating institutions and their specific contributions to each data set are not disclosed. Data within the TriNetX platform are curated and standardized according to established healthcare coding classifications and terminologies, such as *the International Classification of Diseases, Tenth Edition* (ICD‐10), *Current Procedural Terminology* (CPT), and RxNorm [16, 17]. Participating institutions provide aggregated data sets, which undergo rigorous quality control measures implemented by TriNetX in collaboration with these organizations [18]. Prescription data are sourced from pharmacy claims and prescribing systems within electronic health records. The TriNetX platform ensures that no patient‐identifiable information is available for analysis; instead, only deidentified data are presented using aggregated counts and statistical summaries. As a federated network, TriNetX does not require individual ethical approval for studies conducted within its platform. Our study adheres to the Strengthening the Reporting of Observational Studies in Epidemiology (STROBE) guidelines.

We identified patients aged 18–89 years undergoing AF ablation over a 24‐year period (between January 1, 2000 and December 31, 2024) within the TriNetX database. Patients were identified using the CPT code 93656 for AF ablation. Baseline characteristics of patients at the time of AF ablation were extracted, including sex, age, relevant comorbidities, and medications. All ICD‐10 codes used to identify these characteristics are summarized in the Supporting Information S1: Appendix 1. The TriNetX database query was last performed on February 11, 2025, with data extracted from 57 healthcare organizations.

### 2.2 Study Outcomes

Healthcare‐recorded symptoms from inpatient or outpatient settings within 31 days of AF ablation were assessed using ICD‐10 coding and classified into major systems: cardiac (comprising chest pain and palpitations), respiratory (dyspnea and cough), gastrointestinal (nausea, vomiting, heartburn, dysphagia, bloating, diarrhea, constipation, and anorexia), neurological (headache, visual disturbance, speech disturbance, and dizziness or giddiness) and urological (urinary retention and dysuria). These symptoms were selected to comprehensively capture the range of postablation symptoms, including those potentially related to arrhythmia recurrence, procedural complications (e.g., vagus nerve injury, transseptal access), autonomic disturbance, or post‐procedural recovery. Outcomes were analyzed per composite major system and by individual symptom components. The healthcare codes used to identify these outcomes are summarized in the Supporting Information S1: Appendix 1. Symptoms recorded on the day of AF ablation were excluded, due to the inability to confirm their occurrence before or following ablation.

Two falsification endpoints (outcomes which are not expected to be different between cohorts) were included, to quantify unmeasured bias and confounding [19]; these were chosen as falls and pneumonia.

### 2.3 Statistics Analysis

Statistical analyses were performed on the TriNetX online research platform. Baseline characteristics are presented as percentages or mean (standard deviation) as appropriate, and were compared using *χ* ^2^ tests for categorical variables and independent‐sample *t‐*tests for continuous variables. Propensity‐score matching (PSM) was performed to balance baseline differences between the two groups. TriNetX uses a built‐in logistic regression algorithm to generate propensity‐scores, followed by greedy nearest neighbor matching with a caliper of 0.1 pooled standard deviations to identify the matched subsets. Female and male patients were 1:1 propensity‐score matched for age, comorbidities, and medications. Incidence rates were calculated for the outcomes of interest at 31 days for female and male patients, and compared using independent‐sample *t*‐tests (with *p* values < 0.05 considered significant). Unless stated otherwise, results are presented for female versus male patients.

## 3 Results

### 3.1 Demographics and Matching

A total of 112 619 patients underwent AF ablation during the study period (female: 39 279; male: 73 340). Baseline patient characteristics are summarized in Table 1, before and after PSM. After PSM, the final cohorts contained 34 622 patients each (69 244 patients in total).

**Table 1:** Patient characteristics before and after propensity score matching.

	Before PSM				After PSM			
	Female (*n* = 39 279)	Male (*n* = 73 340)	*p* value	SMD	Female (*n* = 34 622)	Male (*n* = 34 622)	*p* value	SMD
Demographics								
Age (years), mean ± SD	67.5 ± 10.1	64.1 ± 10.8	**< 0.001**	0.33	66.9 ± 10.2	66.8 ± 9.9	0.75	0.01
Co‐morbidities (%)								
Paroxysmal AF	70.7	64.8	**< 0.001**	0.13	68.6	68.1	0.15	0.01
Ischemic heart disease	33.4	40.1	< **0.001**	0.14	33.4	32.5	**0.01**	0.02
Hypertension	62.6	61.1	< **0.001**	0.03	60.4	59.3	**0.01**	0.02
Systolic heart failure	12.1	17.2	< **0.001**	0.15	12.4	11.9	0.06	0.02
Diastolic heart failure	16.1	10.6	< **0.001**	0.16	13.6	13.0	**0.02**	0.02
Type 2 diabetes mellitus	18.6	19.2	**0.01**	0.02	17.9	17.0	**0.01**	0.02
Asthma	14.5	7.9	< **0.001**	0.21	11.6	11.3	0.20	0.01
COPD	9.4	8.1	< **0.001**	0.05	8.5	8.3	0.28	0.01
Smoking history	17.4	20.1	< **0.001**	0.07	17.3	16.8	0.09	0.01
GORD	31.0	23.0	< **0.001**	0.18	27.4	26.3	**0.01**	0.02
Gastritis/duodenitis	6.7	4.4	< **0.001**	0.10	5.5	5.4	0.33	0.01
Diverticular disease	13.4	11.4	< **0.001**	0.06	12.2	11.7	0.06	0.01
Irritable bowel syndrome	4.6	1.5	< **0.001**	0.18	2.8	2.7	0.35	0.01
Cerebral infarction	5.7	4.7	< **0.001**	0.05	5.2	5.0	0.22	0.01
Headache	14.3	8.0	< **0.001**	0.20	11.3	11.1	0.14	0.01
Chronic kidney disease	12.7	13.1	0.05	0.01	12.1	11.4	**0.01**	0.02
Obesity	26.2	23.7	**< 0.001**	0.06	24.4	23.5	**0.01**	0.02
Hypothyroidism	22.3	8.2	< **0.001**	0.40	15.1	15.1	0.99	< 0.001
Hyperthyroidism	3.1	1.4	< **0.001**	0.12	2.2	2.1	0.12	0.01
Medications (%)								
Beta‐blocker	76.3	73.2	**< 0.001**	0.07	74.5	73.6	**0.01**	0.02
Flecainide	20.3	14.6	< **0.001**	0.15	18.7	18.5	0.41	0.01
Amiodarone	25.8	27.1	< **0.001**	0.03	25.4	24.5	**0.01**	0.02
Anti‐anginal medication	25.2	25.9	**0.01**	0.02	24.1	23.3	**0.01**	0.02
Antacids	57.6	56.0	< **0.001**	0.03	55.6	54.8	**0.04**	0.02
Omeprazole	24.0	20.0	< **0.001**	0.10	22.1	21.4	**0.03**	0.02
Laxatives	49.2	43.9	< **0.001**	0.11	46.2	44.9	**0.01**	0.03

*Note:* Bold values indicate statistically significant.

Abbreviations: AF, atrial fibrillation; COPD, chronic obstructive pulmonary disease; GORD, gastro‐oesphageal reflux disease; PSM, propensity score matching; SD, standard deviation; SMD, standardized mean difference.

Before PSM, female patients were older (female: 67.5 ± 10.1 vs. male: 64.1 ± 10.8 years; *p* < 0.001) and more often had paroxysmal AF (70.7% vs. 64.8%; *p* < 0.001), with higher rates of most comorbidities including diastolic heart failure (16.1% vs. 10.6%: *p* < 0.001), asthma (14.5% vs. 7.9%; *p* < 0.001), gastro‐esophageal reflux disease (31.0% vs. 23.0%; *p* < 0.001), diverticular disease (13.4% vs. 11.4%; *p* < 0.001), irritable bowel syndrome (4.6% vs. 1.5%; *p* < 0.001), headache (14.3% vs. 8.0%; *p* < 0.001), hypothyroidism (22.3% vs. 8.2%; *p* < 0.001) and hyperthyroidism (3.1% vs. 1.4%; *p* < 0.001). Female patients were more likely to receive beta‐blocker (76.3% vs. 73.2%; *p* < 0.001) and flecainide therapy (20.3% vs. 14.6%; *p* < 0.001), whilst male patients were more likely to receive amiodarone (25.8% vs. 27.1%; *p* < 0.001). Following PSM, female and male patients were well‐matched for age, cardiovascular comorbidities and medications, with standardized mean differences (SMDs) of < 0.1 for all characteristics (Table 1).

### 3.2 Symptoms by Major System

Before PSM, female patients had a higher incidence of cardiac symptoms (9.4% vs. 6.3%; *p* < 0.001), respiratory symptoms (8.5% vs. 6.0%; *p* < 0.001), gastrointestinal symptoms (3.7% vs. 2.1%; *p* < 0.001) and neurological symptoms (3.5% vs. 2.3%; *p* < 0.001) within 31 days of AF ablation compared with male patients. Urological symptoms, however, were more common in male patients (1.0% vs. 1.3%; *p* < 0.001). These differences persisted after PSM, with more cardiac (8.9% vs. 6.1%; *p* < 0.001), respiratory (7.9% vs. 6.1%; *p* < 0.001), gastrointestinal (3.4% vs. 2.2%; *p* < 0.001) and neurological (3.1% vs. 2.5%; *p* < 0.001) symptoms in women, and more urological symptoms in men (0.9% vs. 1.6%; *p* < 0.001) (Table 2).

**Table 2:** Symptoms by major system within 31 days of AF ablation, before and after propensity score matching.

	Before PSM			After PSM		
	Female (*n* = 39 279)	Male (*n* = 73 340)	*p* value	Female (*n* = 34 622)	Male (*n* = 34 622)	*p* value
Cardiac symptoms	9.4%	6.3%	**< 0.001**	8.9%	6.1%	**< 0.001**
Respiratory symptoms	8.5%	6.0%	**< 0.001**	7.9%	6.1%	**< 0.001**
Gastrointestinal symptoms	3.7%	2.1%	**< 0.001**	3.4%	2.2%	**< 0.001**
Neurological symptoms	3.5%	2.3%	**< 0.001**	3.1%	2.5%	**< 0.001**
Urological symptoms	1.0%	1.3%	**< 0.001**	0.9%	1.6%	**< 0.001**

*Note:* Bold values indicate statistically significant.

Abbreviation: PSM, propensity score matching.

Outcomes per major system are summarized in the Central Illustration 1.

**Central Illustration: 1:**
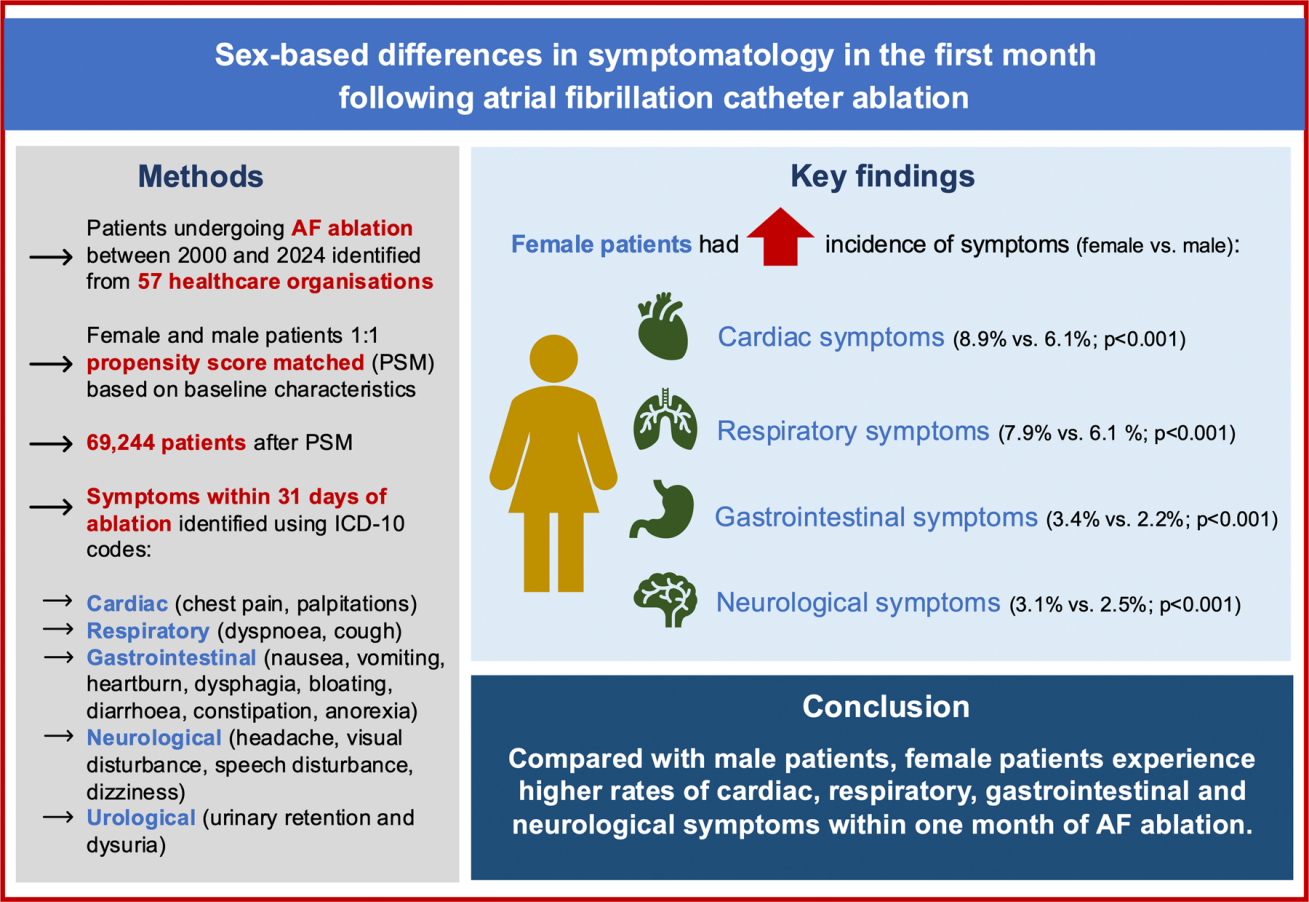
Sex‐based differences in symptomatology in the first month following atrial fibrillation catheter ablation.

### 3.3 Individual Symptom Components

Individual symptom components after PSM are summarized in Table 3. In terms of cardiac and respiratory symptoms, women experienced higher rates of all individual symptom components, including chest pain (5.8% vs. 4.2%; *p* < 0.001), palpitations (4.0% vs. 2.3%; *p* < 0.001), dyspnea (6.6% vs. 5.2%; *p* < 0.001) and cough (1.9% vs. 1.5%; *p* < 0.001). The majority of gastrointestinal symptoms were more common in women, with higher rates of nausea (0.7% vs. 0.3%; *p* < 0.001), vomiting (0.6% vs. 0.3%; *p* < 0.001), dysphagia (0.6% vs. 0.4%; *p* < 0.001), diarrhea (0.8% vs. 0.5%; *p* < 0.001) and constipation (1.1% vs. 0.8%; *p* < 0.001); heartburn (0.1% vs. 0.1%; *p* = 0.49) and anorexia (0.1% vs. 0.1%; *p* = 0.79) were comparable between women and men. From a neurological perspective, women had higher rates of dizziness (1.8% vs. 1.4%; *p* < 0.001) and headache (0.9% vs. 0.7%; *p* < 0.001), although no difference in visual disturbance (0.6% vs. 0.5%; *p* = 0.31) or speech disturbance (0.2% vs. 0.2%; *p* = 0.51). Rates of urinary retention were higher in men (0.3% vs. 1.1%; *p* < 0.001), with no difference in dysuria (0.6% vs. 0.05%; *p* = 0.06).

**Table 3:** Individual symptom outcomes within 31 days of AF ablation after propensity score matching.

	After PSM		
	Female (*n* = 34 622)	Male (*n* = 34 622)	*p* value
Chest pain	5.8%	4.2%	**< 0.001**
Palpitations	4.0%	2.3%	**< 0.001**
Dyspnea	6.6%	5.2%	**< 0.001**
Cough	1.9%	1.5%	**< 0.001**
Nausea	0.7%	0.3%	**< 0.001**
Vomiting	0.6%	0.3%	**< 0.001**
Heartburn	0.1%	0.1%	0.49
Dysphagia	0.6%	0.4%	**< 0.001**
Bloating	0.2%	0.2%	> 0.99
Diarrhea	0.8%	0.5%	**< 0.001**
Constipation	1.1%	0.8%	**< 0.001**
Anorexia	0.1%	0.1%	0.79
Headache	0.9%	0.7%	**< 0.001**
Visual disturbance	0.6%	0.5%	0.31
Speech disturbance	0.2%	0.2%	0.51
Dizziness or giddiness	1.8%	1.4%	**< 0.001**
Urinary retention	0.3%	1.1%	**< 0.001**
Dysuria	0.6%	0.5%	0.06

*Note:* Bold values indicate statistically significant.

Abbreviation: PSM: propensity score matching.

### 3.4 Falsification Endpoints

There was no difference in the falsification endpoints between female and male patients after PSM (falls: female, 0.4% vs. male, 0.3%, *p* = 0.15; pneumonia: female 0.8% vs. 0.9%, *p* = 0.19).

## 4 Discussion

This study is, to our knowledge, the first to describe sex‐based differences in clinical symptoms in the month following AF ablation. The primary findings from our analysis are: (i) In an unselected cohort of patients undergoing AF ablation, women experienced higher rates of cardiac, respiratory, gastrointestinal and neurological symptoms within 31 days of AF ablation, whilst men experienced higher rates of urological symptoms; (ii) These differences persisted despite propensity score matching for baseline characteristics, suggesting female sex is an independent predictor of adverse symptoms immediately following ablation (Central Illustration 1).

Below, we discuss the clinical relevance of these findings and potential solutions to reducing these differences.

### 4.1 Sex‐Based Differences in Symptoms Postablation

Multiple studies have shown that women are more symptomatic from AF and have lower quality of life than men [12, 20, 21]. Possible explanations for this include delay in diagnosis and treatment, increased comorbidity burden, higher resting heart rate, higher adverse events from antiarrhythmic drug therapy, and higher levels of psychological distress [7, 12, 22, 23]. Further, observational studies suggest that women are less likely to receive invasive rhythm control therapy—including electrical cardioversion or ablation—compared with men [24, 25]. Data on sex‐specific outcomes following AF catheter ablation are conflicting. For example, observational studies have reported a higher risk of AF recurrence and adverse events in women [10, 11], whilst two large randomized controlled trials (CABANA [9] and CIRCA‐DOSE [8]) found no relevant difference between sexes in terms of efficacy or safety. Potential reasons for disparities in procedural outcomes include smaller left atria with thinner walls, higher occurrence of non‐pulmonary vein triggers, and more extensive atrial fibrosis in women [26, 27, 28]. Crucially, sex‐specific data on early post‐procedural recovery and symptoms are lacking.

The findings of our analysis suggest that women with AF experience a higher symptom burden in the first month postablation, aligning with previous research indicating greater preprocedural symptom severity. Whilst our analysis cannot fully determine the reasons behind this difference, a possible explanation is that women often present with more advanced symptomatic disease by the time they undergo ablation. However, this alone is unlikely to account for the entire difference, particularly since symptom disparities persisted after adjustment for baseline characteristics.

Other contributing factors may include heightened autonomic sensitivity in women [11, 29], differences in inflammatory responses [30], and variations in pain perception between sexes [31]. The higher prevalence of anxiety and depression among women with AF may further amplify symptom reporting [22, 32], though this remains speculative. Furthermore, prior work using continuous rhythm monitoring has shown that women with paroxysmal AF are more likely than men to report cardiac symptoms in the absence of documented AF [33]. This suggests that sex differences in symptom perception and attribution may also contribute to the observed difference, independent of arrhythmia burden [33]. Regardless of the underlying mechanisms, these findings are significant, as they highlight the need for a more tailored approach to postablation care in women. Interestingly, men experienced higher rates of urological symptoms postablation, which may reflect differences in procedural factors such as urethral catheterization, although specific data on catheter use were not available in our analysis.

Our analysis focused on symptoms within 1 month of ablation, as this period carries the highest risk for post‐procedural symptoms, particularly those relating to vagus nerve injury. A study by Jacobs et al. assessed gastrointestinal symptoms in 100 patients undergoing radiofrequency ablation for AF using questionnaires. Symptoms rated as moderate were common at baseline, increased at 1‐month postablation, and returned to baseline by 3 months [34]. Notably, in clinical practice, many patients have their first postablation review at 3 months, meaning symptoms before this point—unless severe—are likely underreported [35]. As clinicians, recognizing the high incidence of these early symptoms, particularly in women, is crucial for patient education and post‐procedural management.

### 4.2 Potential Solutions to Reducing Disparities

Addressing sex‐based disparities in AF ablation outcomes requires a comprehensive approach. A crucial initial step is increasing awareness among healthcare professionals regarding the delayed referral of women for AF ablation. Indeed, earlier intervention may prevent disease progression and reduce preprocedural symptom severity, which could, in turn, lower postablation symptom burden. Further, optimizing ablation techniques for women is key area for improvement. For example, unlike disparities observed using thermal ablation modalities (radiofrequency and cryoablation) [9, 11], pulsed field ablation (PFA) appears to perform similarly between sexes [36]. In the European multi‐centre MANIFEST registry, women experienced similar 1‐year freedom from atrial arrhythmia (female: 79% vs. male: 76.3%; *p* = 0.28) with no significant difference in major adverse events (2.5% vs. 1.5%; *p* = 0.19) [36]. Alongside this, the cardioselectivity and short procedural times [37, 38] associated with PFA may help reduce post‐procedural symptoms across both sexes. More widely, ensuring adequate representation of women in AF ablation trials is essential for developing evidence‐based guidelines that reflect sex‐specific differences in AF pathophysiology and treatment response. Future studies should seek to assess different ablation modalities and settings to account for anatomical differences between men and women, to improve both safety and efficacy.

Beyond this, postablation care should be tailored to better address early symptoms. Enhanced pre‐ and post‐procedural patient education, closer follow‐up, and structured symptom management protocols may help mitigate symptom amplification. Proactive symptom management through individualized medication adjustments (e.g., analgesics or anti‐emetics) could further improve patient experience and satisfaction. Of note, the current holistic or integrated care approach to AF management recommended in global guidelines emphasizes the need for patient‐centered, symptom‐directed decisions on rhythm or rate control, in conjunction with stroke prevention and attention to comorbidities [39, 40, 41, 42, 43]. Such a patient‐centred holistic management approach has been associated with improved clinical outcomes in different healthcare settings [44, 45, 46].

### 4.3 Limitations

Our study has several limitations. First, as a retrospective observational analysis, our findings are limited by the quality and completeness of electronic health record data. Although the TriNetX database provides access to a large, diverse patient cohort, reliance on ICD‐10 codes for symptom identification may introduce misclassification bias. Second, the database provides only symptoms reported to and recorded by healthcare institutions; some patients may have experienced symptoms within 1 month of ablation but not sought medical advice during this time. Some symptoms may also have predated the procedure. Additionally, because symptom entries were not linked to concurrent rhythm data, we cannot determine whether symptoms occurred during episodes of arrhythmia or in sinus rhythm. Despite this, our findings remain relevant, as they capture real‐world healthcare‐reported symptoms. Third, ablation modality (radiofrequency, cryoablation, or PFA) and extent (e.g., pulmonary vein isolation only vs. additional lesions) were not available for analysis; it is possible that these factors may influence post‐procedural symptom profiles and could contribute to sex‐based differences in outcomes. Fourth, data on postablation medication usage and management protocols were not available, limiting our ability to assess their impact on symptom reporting and resolution. Fifth, to preserve patient and centre anonymity, the geographical distribution of patients was not available for analysis. Finally, while PSM was employed to balance baseline differences between cohorts, and falsification endpoints were negative, residual confounding may still be present.

## 5 Conclusion

This study highlights sex‐based differences in early post‐procedural symptoms following AF ablation, with women experiencing higher rates of cardiac, respiratory, gastrointestinal, and neurological symptoms. Addressing these disparities through earlier referral for ablation, tailored post‐procedural management protocols, and optimization of ablation techniques should be prioritized in future clinical practice and research studies.

## Conflicts of Interest

G.Y.H.L. is a consultant and speaker for BMS/Pfizer, Boehringer Ingelheim, Daiichi‐Sankyo, Anthos. No fees are received personally. He is a National Institute for Health and Care Research (NIHR) Senior Investigator and co‐PI of the AFFIRMO project on multimorbidity in AF (grant agreement No 899871), TARGET project on digital twins for personalized management of atrial fibrillation and stroke (grant agreement No 101136244) and ARISTOTELES project on artificial intelligence for management of chronic long term conditions (grant agreement No 101080189), which are all funded by the EU's Horizon Europe Research and Innovation programme. D.G. reports institutional research grants from Biosense Webster, Boston Scientific and Medtronic, and speaker fees from Boston Scientific. The other authors declare no conflicts of interest.

## Supporting information

Supporting material JCE.

## Data Availability

The data that support the findings of this study are available from TriNetX. Restrictions apply to the availability of these data, which were used under license for this study. Data are available from https://trinetx.com/with the permission of TriNetX.
