# Supplementary material for: Sex‐Based Differences in Symptomatology in the First Month Following Atrial Fibrillation Catheter Ablation
Source: J Cardiovasc Electrophysiol. 2025 Jul 11;36(9):2271–8. doi: 10.1111/jce.70009 (PMC12420893; doi:10.1111/jce.70009)
Supplement: Supplementary file 1 — Supporting material JCE. [file JCE-36-2271-s001.docx]

**Supplementary Materials – Appendix 1: Diagnostic codes**

*Abbreviations used below:*

- *ICD10CM: International Classification of Diseases, Tenth Revision, Clinical Modification*
- *CPT: Current Procedural Terminology*

**Diagnostic codes for inclusion and baseline characteristics**

**Atrial fibrillation catheter ablation:**

- CPT:93656 (Comprehensive electrophysiologic evaluation including transseptal catheterizations, insertion and repositioning of multiple electrode catheters with intracardiac catheter ablation of atrial fibrillation by pulmonary vein isolation, including intracardiac electrophysiologic 3-dimensional mapping, intracardiac echocardiography including imaging supervision and interpretation, induction or attempted induction of an arrhythmia including left or right atrial pacing/recording, right ventricular pacing/recording, and His bundle recording, when performed)

**Baseline characteristics:**

- Paroxysmal atrial fibrillation: ICD10CM:I48.0 (Paroxysmal atrial fibrillation)
- Ischaemic heart disease: ICD10CM:I20-I25 (Ischemic heart diseases)
- Hypertension: ICD10CM:I10 (Essential (primary) hypertension
- Systolic heart failure: ICD10CM:I50.2 (Systolic (congestive) heart failure)
- Diastolic heart failure: ICD10CM:I50.3 (Diastolic (congestive) heart failure)
- Type 2 diabetes mellitus: ICD10CM:E11 (Type 2 diabetes mellitus)
- Asthma: ICD10CM:J45 (Asthma)
- Chronic obstructive pulmonary disease: ICD10CM:J44.9 (Chronic obstructive pulmonary disease, unspecified)
- Smoking history: ICD10CM:Z87.891 (Personal history of nicotine dependence)
- Gastro-oesophageal reflux disease: ICD10CM:K21 (Gastro-esophageal reflux disease)
- Gastritis/duodenitis: ICD10CM:K29 (Gastritis and duodenitis)
- Diverticular disease: ICD10CM:K57 (Diverticular disease of intestine)
- Irritable bowel syndrome: ICD10CM:K58 (Irritable bowel syndrome)
- Cerebral infarction: ICD10CM:I63 (Cerebral infarction)
- Headache: ICD10CM:R51 (Headache)
- Chronic kidney disease: ICD10CM:N18 (Chronic kidney disease)
- Obesity: ICD10CM:E66.9 (Obesity, unspecified)
- Hypothyroidism: ICD10CM:E03.9 (Hypothyroidism, unspecified)
- Hyperthyroidism: ICD10CM:E05.9 (Thyrotoxicosis, unspecified)

**Medications:**

- Beta-blocker: Veterans Affairs CV100 (Beta blockers/related)
- Flecainide: RxNorm 4441 (Flecainide)
- Amiodarone: RxNorm 703 (Amiodarone)
- Anti-anginal medication: Veterans Affairs CV250 (Antianginals)
- Antacids: Veterans Affairs GA100 (Antacids)
- Omeprazole: RxNorm 7646 (Omeprazole)
- Laxatives: Veterans Affairs GA200 (Laxatives)

**Diagnostic codes for outcomes**

**Cardiac symptoms:**

- Chest pain: ICD10CM:R07.1 (Chest pain on breathing), or ICD10CM:R07.2 (Precordial pain), or ICD10CM:R07.8 (Other chest pain), or ICD10CM:R07.9 (Chest pain, unspecified)
- Palpitations: ICD10CM:R00.2 (Palpitations)

**Respiratory symptoms:**

- Dyspnoea: ICD10CM:R06.0 (Dyspnea)
- Cough: ICD10CM:R05 (Cough)

**Gastrointestinal symptoms:**

- Nausea: ICD10CM:R11.0 (Nausea)
- Vomiting: ICD10CM:R11.2 (Nausea with vomiting, unspecified)
- Heartburn: ICD10CM:R12 (Heartburn)
- Dysphagia: ICD10CM:R13 (Aphagia and dysphagia)
- Bloating: ICD10CM:14.0 (Abdominal distension (gaseous)
- Diarrhoea: ICD10CM:R19.7 (Diarrhea, unspecified)
- Constipation: ICD10CM:K59.0 (Constipation)
- Anorexia: ICD10CM:R63.0 (Anorexia)

**Neurological symptoms:**

- Headache: ICD10CM:R51 (Headache)
- Visual disturbance: ICD10CM:H53 (Visual disturbances)
- Speech disturbance: ICD10CM:R47 (Speech disturbance, not elsewhere classified)
- Dizziness or giddiness: ICD10CM:R42 (Dizziness and giddiness)

**Urological symptoms:**

- Urinary retention: ICD10CM:R33 (Retention of urine)
- Dysuria: ICD10CM:R30.0 (Dysuria)

**Falsification endpoints:**

- Falls: ICD10CM:W00-W19 (Slipping, tripping, stumbling and falls)
- Pneumonia: ICD10CM:J.18 (Pneumonia, unspecified organism)
